# The Emerging Roles of Human Gut Microbiota in Gastrointestinal Cancer

**DOI:** 10.3389/fimmu.2022.915047

**Published:** 2022-06-15

**Authors:** Qianqian Guo, Hai Qin, Xueling Liu, Xinxin Zhang, Zelong Chen, Tingting Qin, Linlin Chang, Wenzhou Zhang

**Affiliations:** ^1^Department of Pharmacy, The Affiliated Cancer Hospital of Zhengzhou University & Henan Cancer Hospital, Zhengzhou, China; ^2^Department of Clinical Laboratory, Guizhou Provincial Orthopedic Hospital, Guiyang City, China; ^3^The Second Clinical Medical School of Nanjing Medical University, Nanjing Medical University, Nanjing, China; ^4^The Affiliated Cancer Hospital of Zhengzhou University & Henan Cancer Hospital, Henan Province Engineering Research Center of Artificial Intelligence and Internet of Things Wise Medical, Zhengzhou, China

**Keywords:** gut microbiota, gastric cancer, colorectal cancer, SCFAs, therapy

## Abstract

The gut microbiota is composed of a large number of microorganisms with a complex structure. It participates in the decomposition, digestion, and absorption of nutrients; promotes the development of the immune system; inhibits the colonization of pathogens; and thus modulates human health. In particular, the relationship between gut microbiota and gastrointestinal tumor progression has attracted widespread concern. It was found that the gut microbiota can influence gastrointestinal tumor progression in independent ways. Here, we focused on the distribution of gut microbiota in gastrointestinal tumors and further elaborated on the impact of gut microbiota metabolites, especially short-chain fatty acids, on colorectal cancer progression. Additionally, the effects of gut microbiota on gastrointestinal tumor therapy are outlined. Finally, we put forward the possible problems in gut microbiota and the gastrointestinal oncology field and the efforts we need to make.

## 1 Introduction

The gut microbiota includes the organisms living in the gastrointestinal tract and is a large and complicated ecosystem. As a rough estimate, the total weight of adult human gut microbiota is 1.5 kg, consisting of 3.9 × 10^13^ microorganisms/ml of luminal content (1). The gut microbiota mainly includes bacteria, fungi, protozoa, archaea, and viruses, among which bacteria are dominant (2). The composition of the human gut microbiota is not homogeneous (3). According to the differences in intestinal pH and oxygen content, bacterial groups were distributed at different locations, and bacterial concentrations increased from stomach to rectum. During early development, the gut microbiota undergoes a systematic turnover of species until a stable adult state is reached (4). Neonates have very dynamic changes in the composition of their gut microbiota (5). Although the composition of the gut microbiota is affected by several factors, such as age, diet, and lifestyle, it is relatively stable in adults under normal physiological conditions (6).

The gut microbiota has a strong metabolic capacity and is deemed an important “metabolic organ,” which plays an important role in host digestion, nutrient absorption, metabolism, immunity, and other processes (3). Recently, with the rapid progression of sequencing technology, gut microbiota has been confirmed to be involved in the occurrence and development of various tumors. In particular, changes in gut microbial metabolites, such as short-chain fatty acids (SCFAs), tryptophan metabolites, and secondary bile acids, may have broad implications for the formation and progression of various tumors (7–9). To our knowledge of the role of the tumor microenvironment in cancer progression and treatment, the impact of the gut microbiota on tumor immunity has obtained increasing attention. Changes in gut microbiota can affect not only tumor immunotherapy but also chemotherapy treatment (10). Therefore, targeting gut the microbiota could serve as a novel therapeutic option.

The relationship between gut microbiota and tumors has become an important issue in multiple studies. In this review, we only focused on the bacteria and reviewed the potential roles of gut microbiota and microbial metabolites in gastrointestinal cancer. In addition, we discussed the therapeutic potential of gut microbiome in gastrointestinal cancer.

## 2 Gut Microbiota and Gastrointestinal Cancer

The carcinogenic process usually consists of three stages: initiation, progression, and metastasis. It can be influenced by the oncogenic effects of gut microbiota and their products, modulating circulatory metabolite levels that might inhibit or promote tumor growth, inducing pro-inflammatory and immunosuppressive effects (11, 12). Below, we focused on bacteria in gastric cancer (GC) and colorectal cancer (CRC).

### 2.1 Gastric Cancer

**Figure 1:**
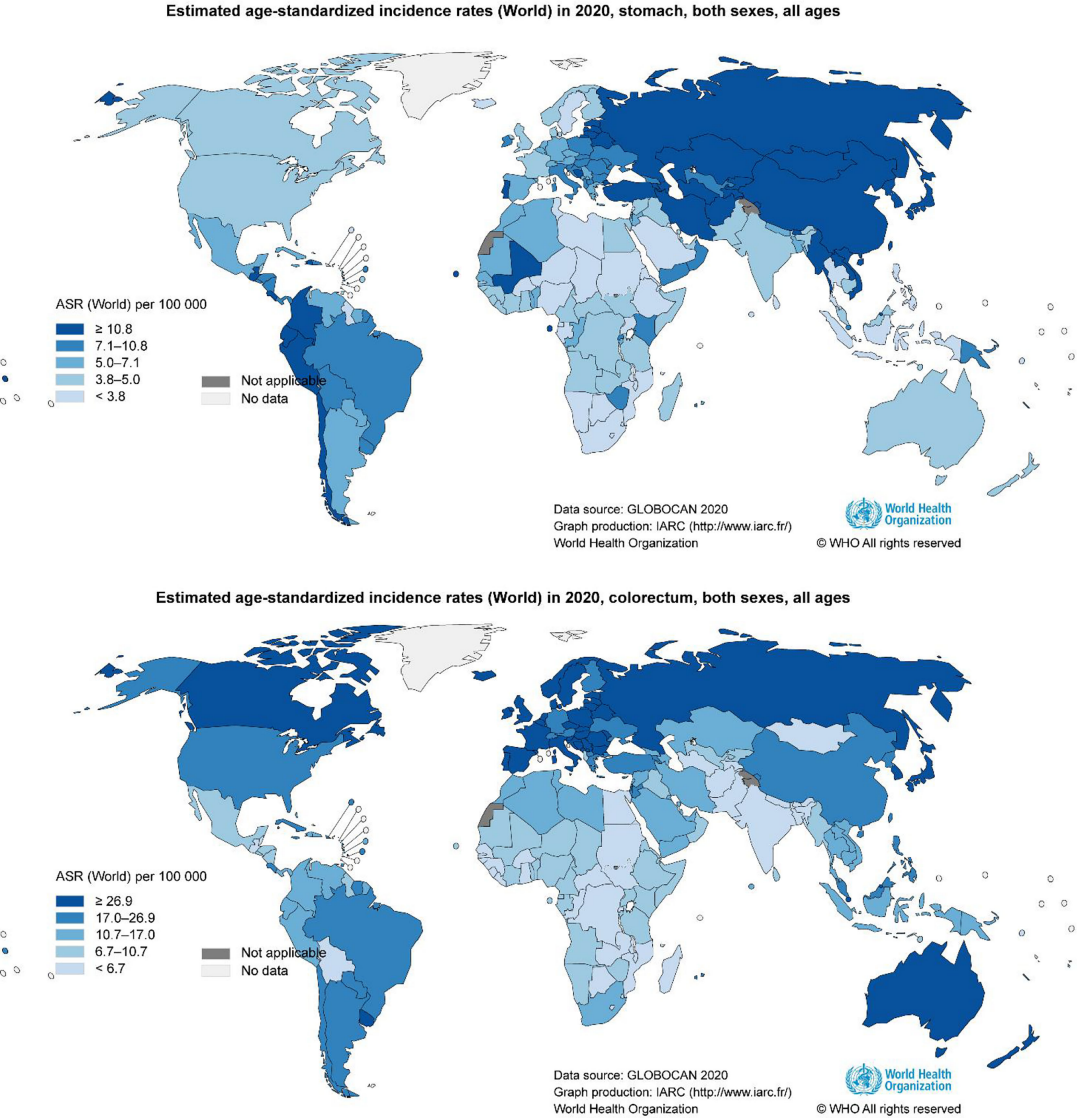
Geographical variation in gastrointestinal cancer rates. Data from the WHO demonstrating the high incidence of gastrointestinal cancer (13).

GC is the fifth most common cancer and the fourth leading cause of cancer-associated death worldwide (13). As illustrated by the latest WHO data, the incidence of GC exhibits significant regional differences (13). East Asia, Eastern Europe, and South America are hotspots for GC incidence and mortality (**Figure 1**). The risk factors for GC are associated with various etiologies, including *Helicobacter pylori* (*H. pylori*) infection, high salt intake, age, and a low fruit and vegetable diet. Chronic infection by *H. pylori* is the most well-described cause of non­cardia GC (14).

#### 2.1.1 *H. pylori*

*H. pylori* is an aerobic Gram-negative bacterium, with catalase, urease, and oxidase activities (15). *H. pylori* has been recognized as a class I carcinogen by the World Health Organization. *H. pylori* infection-induced GC is associated with bacterial virulence, host genetic polymorphism, and environmental factors (14). *H. pylori* infection increases gastric pH, changes the composition of gastric microbiota, and then creates favorable niches for bacterial colonization.

*H. pylori* possibly directly affects carcinogenesis by vacuolating cytotoxin A (VacA) and cytotoxin-associated gene A (CagA) (16). On the one hand, all *H. pylori* strains contain a single chromosomal *VacA* gene, encoding a 140 kDa VacA protein, which is an intracellular-acting and channel-forming toxin. Through impairing host endolysosomal trafficking, VacA induces the accumulation of dysfunctional lysosomes and autophagosomes (17). Additionally, VacA can increase reactive oxygen species, mitochondrial damage, and inflammation (18). Furthermore, VacA may attenuate the host immune response, thereby facilitating persistent *H. pylori* colonization in the stomach (18). However, most *H. pylori* strains possess a *CagA* pathogenicity island encoding a 120–140 kDa CagA protein, which is a strain-specific protein and is transferred into host cells by the type IV secretion system. CagA inhibits the apoptotic pathway of epithelial cells and causes morphological aberrations, namely, loss of cell polarity and adhesion, increased cell motility, scattering, and elongation (19). Additionally, CagA is deemed a key oncogene in human chronic gastritis and gastric ulcer, MALT lymphoma, and GC (20).

Although *H. pylori* induces chronic gastritis and peptic and duodenal ulcers and is linked to more than 90% of GC cases, only approximately 1 to 3% of infected individuals progress to GC (21–23). During a 7.5-year follow-up randomized controlled trial, Wong et al. did not find a distinctive benefit of *H. pylori* eradication when compared with placebo treatment (24). However, in the subgroup of *H. pylori* carriers without precancerous lesions, Wong et al. found that eradication treatment of *H. pylori* significantly decreased GC progression (24). In a 5-year prospective study of 1,755 patients, Rugge et al. confirmed that eradication treatment of *H. pylori* did not abolish the risk of neoplastic progression in subjects with advanced stages (III–IV) (25). Consistently, eradication of *H. pylori* was shown to still be effective in a subset of early GC patients by minimizing the risk of metachronous GC (26, 27). Furthermore, Guo et al. suggested that *H. pylori* eradication treatment could restore the gastric microbiota to a similar status as negative subjects and may exert more beneficial effects on the gut microbiota, such as downregulation of drug-resistance and probiotic enrichment (28). Thus, *H. pylori* eradication treatment seems to counteract the risk of GC transformation, but the magnitude depends on the degree of pre­existing damage at the time of eradication.

#### 2.1.2 Non-*H. pylori* Gastric Bacteria

With the application of high throughput sequencing technology and metagenomics in microbiology, other acid-resistant bacteria have been found in the stomach besides *H. pylori*. Nasr et al. revealed the major studies in which non-*H. pylori* have been implicated in GC development before 2019 (20). Thus, we summarized the association between *non-H. pylori* and GC progression over the past five years, especially in the past three years. In summary, the limited data available to date showed that the bacterial genera that were most invariably reported to be enriched in GC patients include *Lactobacillus*, *Streptococcus*, *Prevotella*, *Veillonella*, and so on (**Table 1**).

**Table 1:** Non-*H. pylori* bacteria in GC.

Study Sample	Tissue	Cancer-associated bacteria Other Than *H. pylori*	Ref.
Gastritis (n = 16) GAD (n = 16) EGC (n = 36) AGC (n = 20)	gastric juice	Phylum level: *Firmicutes* was the most dominant taxa in all groups; Genus level, *Streptococcus* was the most dominant taxa in all groups; enriched in GAD: *Patescibacteria*, *Saccharimonadaceae*, *Granulicatella* and *Veillonella* enriched in AGC: *Veillonella*, *Alloprevotella* and *Lactobacillus*.	Park et al. (29)
EGC (n = 4) IM (n = 17) mild IM (n = 16) multifocal IM (n = 6)	gastric antral biopsies	GC carcinogenesis stages were represented by enrichment of *Proteobacteria* and depletion of *Bacteroidetes* enriched in EGC: *Proteus* genus, *Moryella* genus, *Phyllobacteriaceae*, *Enhydrobacter* and *Lactobacillus*.	Png et al., 2022 (30)
HC (n = 27) GC (n = 43)	gastric antrum; gastric cancer; adjacent noncancerous	all alpha-diversity indices were higher in GC; enriched in GC: *Pasteurellaceae* and *Enterococcaceae*.	Park et al. (31)
GC (n = 37)	tumor tissues and matched non-tumor tissues	phylum level enriched in GC: *Firmicutes*, *Bacteroidetes*, *Actinobacteria*, *Fusobacteria*, and *Spirochetes*. genus level enriched in GC: *Lactobacillus*, *Streptococcus*, *Acinetobacter*, *Prevotella*, *Sphingomonas*, *Bacteroides*, *Fusobacterium*, *Comamonas*, *Empedobacter*, and *Faecalibacterium*.	Dai et al. (32)
HC (n = 25) IM (n = 18) EGC (n = 34)	Endoscopic biopsies from antrum and corpus	enriched in GC: *Firmicutes*, *Gemella* and *Streptococcus*. decreased in GC: *Proteobacteria*.	Pimentel-Nunes et al. (33)
GC (n = 18) SG (n = 32)	Paired tumor and paracancerous samples of the gastric mucosa	enriched in GC: *Dialister*, *Helicobacter*, *Lactobacillus*, *Rhodococcus*, *Rudaea* and *Sediminibacterium*.	Wu et al. (34)
SG (n = 25) GC (n = 34)	Antrum corpus	Enriched in GC: *Proteobacteria*, *Firmicutes*, *Actinobacteria* and *Bacteroidetes*	Deng et al. (35)
SRCC (n = 10) GAD (n = 10)	formalin-fixed paraffin-embedded GC samples	enriched in SRCC: *Fusobacteria*, *Bacteroidetes*, and *Patescibacteria*. enriched in GAD: *Proteobacteria* and *Acidobacteria phyla*	Ravegnini et al. (36)
HC (n = 30) non-atrophic CG (n = 21) IM(n = 27) IN(n = 25) GC (n = 29)	gastric mucosal biopsy	enriched in GC: *Actinobacteria*, *Bacteriodes*, *Firmicutes*, and *Fusobacteria*.	Wang et al. (37)
GC patients without preoperative chemotherapy(n = 276)	Tumoral (n=229) Peritumoral (n=247) normal tissues(n=230)	enriched in tumoral microhabitat: *Streptococcus*, *Selenomonas*, *Fusobacterium*, *Propionibacterium*, and *Corynebacterium*.	Liu et al. (38)
SG (n = 77) AG (n = 74) IM (n = 17) GC (n = 39)	Antrum; cancer lesions body and fundus for SG, AG and IM; adjacent non-cancerous tissues for GC;	enriched in GC: Oral bacteria, *Peptostreptococcus*; *Streptococcus anginosus*; *Slackia*, *Gemella* and *Fusobacterium*.	Coker et al. (39)

Numerous studies have shown that probiotics, including lactic acid bacteria (LAB), can enhance gastrointestinal health, immune regulation, and cancer prevention (40). Even though most of the aforementioned bacteria implicated in GC are LAB, including *Lactobacillus*, *Streptococcus*, and *Lactococcus*. A previous study has confirmed that LAB can induce the generation of reactive oxygen species (41) which initiates cancer angiogenesis, metastasis, and survival under individual concentrations (42). In addition, LAB have been proved to form large number of N-nitroso compounds (43, 44), which promote angiogenesis, mutagenesis, protooncogene expression, and inhibit apoptosis (45–47). Furthermore, LAB can elevate lactate production, which is crucial for major carcinogenic processes, namely, cell migration, angiogenesis, metastasis, immune evasion, and cell sufficiency (48, 49).

### 2.2 Colorectal Cancer (CRC)

**Figure 2:**
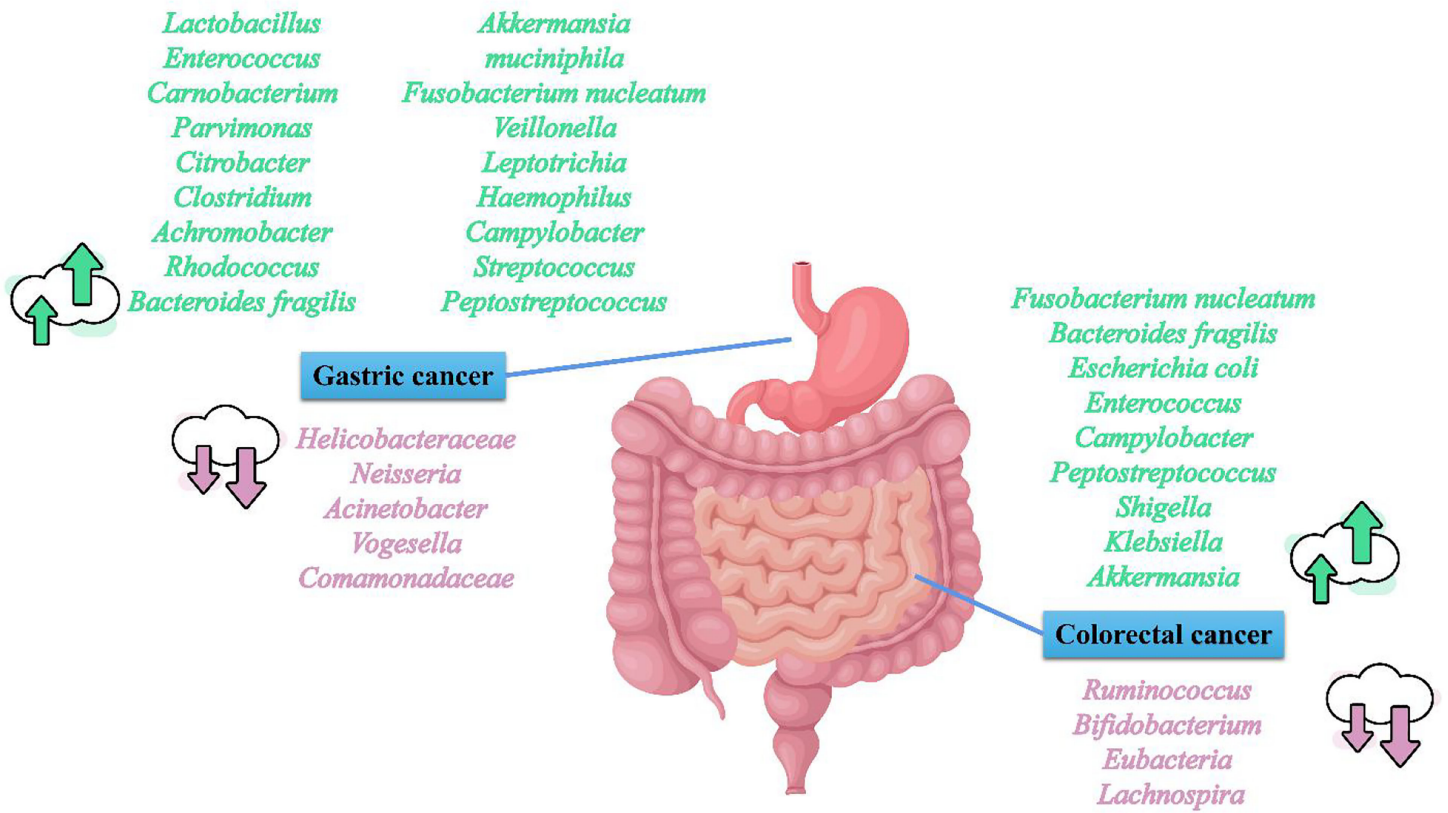
Overview on microbiota and gastrointestinal cancer.

CRC is the third most common cancer and the second leading cause of cancer-associated deaths worldwide (13). Like GC, CRC incidence also displays regional differences (13). Russia, Canada, and Australia are hotspots for CRC incidence and mortality (**Figure 1**). CRC is constantly related to the changes in the microbial composition of the tumor and its adjacent mucosa, which is termed as dysbiosis (50–54). Dysbiosis is characterized by the expansion of the bacterial taxa to a certain extent. However, the dominant bacterial species in CRC evolution are still unknown. Several studies have reported higher proportions of *Fusobacterium nucleatum* (*F. nucleatum*), *Bacteroides fragilis* (*B. fragilis*), *Escherichia coli* (*E. coli*), *Enterococcus*, *Campylobacter*, *Peptostreptococcus*, *Shigella*, *Klebsiella*, and *Akkermansia* in CRC patients, and lower levels of *Ruminococcus*, *Bifidobacterium*, *Eubacteria*, and *Lachnospira* compared with healthy subjects (55–64) (**Figure 2**). Although *Akkermansia* has been shown to be a potential probiotic of the new generation and plays critical roles in obesity, diabetes, and atherosclerosis (65), its effects in CRC progression are still confusing. Furthermore, since *F. nucleatum*, *B. fragilis*, and *E. coli* are widely shown to play an important role on CRC development,we here focused on *F. nucleatum*, *B. fragilis*, and *E. coli*, and subsequently elucidated the correlation between gut microbiota and CRC progression.

#### 2.2.1 *F. nucleatum*

*F. nucleatum*, a gram-negative anaerobe, which is universal in the oral cavity, is absent or rarely detected elsewhere in the body under normal conditions (66). Recently, *F. nucleatum* was found to be enriched in CRC and the rectal swabs of CRC patients (66–69), for example, Castellarin et al. verified *F. nucleatum* overabundance in tumor tissue, and observed a positive correlation with lymph node metastasis (68); Mima et al. confirmed that the amount of *F. nucleatum* in CRC tissues were associated with a shorter survival (70); Consistently, Yan et al. analyzed *F. nucleatum* level and prognostic significance in CRC patients, and found that the *F. nucleatum* level was abnormally high in tumor tissues and correlated with tumor invasion, lymph node metastasis status, and distant metastasis (71).

In addition, a correlation between *F. nucleatum* and CRC has also been found in preclinical experiments. Rubinstein et al. demonstrated that *F. nucleatum* promoted CRC cell progression by modulating the E-cadherin/B-catenin signaling through its unique FadA adhesin, and FadA expression was correlated with the expression of oncogenic and inflammatory genes in CRC patients (72). Kostic et al. indicated that, in the Apc (Min/+) mouse model of intestinal tumorigenesis, *F. nucleatum* increased tumor multiplicity and selectively recruited tumor-infiltrating myeloid cells, which can promote tumor progression (73); Yang et al. found that *F. nucleatum*-infected CRC cells exhibited an increased proliferation, invasive activity, and ability of tumor formation (74). Furthermore, *F. nucleatum*-activated Toll-like receptor 4 signaling to MYD88 activated nuclear factor-κB and thus increased miR-21 expression, and miR-21 could subsequently reduce the expression of RASA1 (74). RASA1 is a member of the RAS GTPase activating proteins (RAS-GAP) family, and mutation or loss of function of RASA1 can activate the RAS-MAPK cascade in CRC (75–77). Moreover, Brennan et al. suggested that *F. nucleatum* influenced intestinal immunity by shaping Th17 responses in an FFAR2-dependent manner (78). Although further studies are necessary to clarify the multifaceted roles of FFAR2, this research highlighted a conserved pathway that could be targeted to attenuate oncomicrobe-mediated CRC. These studies are consistent with the results in clinical studies that *F. nucleatum* is closely related to CRC, but further mechanisms between *F. nucleatum* and CRC still require more evidence.

#### 2.2.2 Enterotoxigenic *B. fragilis* (ETBF)

*B. fragilis*, an anaerobic commensal that constitutes only 1–2% of the gut microbiota, can trigger diarrhea and inflammatory bowel disease by producing *B. fragilis* toxin (79, 80). *B. fragilis* phylogeny can be described in multiple ways and categorized as toxigenic or non-toxigenic (81). For example, ETBF is associated with inflammatory diseases and CRC. For example, sero-positivity of *B. fragilis* and *E. coli* was associated with CRC development, suggesting that co-infection of these bacterial species contributes to CRC tumorigenesis (82). Boleij et al. showed that the *B. fragilis* toxin gene was associated with CRC, especially in late-stage CRC (83). In addition, several experimental evidence has implicated enterotoxigenic ETBF in CRC development, like Liu et al. found that ETBF upregulated JMJD2B levels in a TLR4-NFAT5-dependent pathway and induced CRC stemness (84); Cao et al. confirmed that ETBF promoted intestinal inflammation and malignancy by downregulating miR-149-3p and further promoting PHF5A-mediated RNA alternative splicing of KAT2A in CRC cells (85); Guo et al. suggested that the downregulation of farnesoid X receptor promoted CRC development by facilitating ETBF colonization (86); Goodwin et al. demonstrated that *B. fragilis* toxin upregulated spermine oxidase, increased reactive oxygen species, and DNA damage, thereby propagating inflammation and tumorigenesis (87).

#### 2.2.3 *E. coli*

*E. coli* is a highly prevalent, but not very abundant, gram-negative facultative anaerobe of the distal gastrointestinal tract. *E. coli* is a vast and diverse group of bacteria. Colibactin-producing *E. coli* are closely related to CRC (88–91). Bonnet et al. observed an increased level of mucosa-associated and internalized *E. coli* in tumors compared with normal tissue, and colonization of mucosa by *E. coli* was associated with poor prognosis in colon cancer (tumor-node-metastasis stage) (88); *E. coli* from the B2 phylogenetic group is implicated in CRC as it possesses a genomic island, termed polyketide synthetase (pks), which codes for the synthesis of colibactin, a genotoxin that induces DNA damage, cell cycle arrest, mutations, and chromosomal instability in eukaryotic cells. In addition, Iyadorai found that pks^+^ *E. coli* was isolated in CRC patients (89). Furthermore, the influence of *E. coli* on CRC was also identified in animal studies, like *E. coli*-increased tumorigenesis in murine models of CRC (92, 93), and Cougnoux et al. found that colibactin-producing *E. coli* enhanced tumor growth in both xenograft and azoxymethane/dextran sodium sulfate models, and tumor growth was sustained by cellular senescence (92). However, note that, in addition to the tumor-facilitating effects, some *E. coli* are commensal and even probiotic (94).

In conclusion, the roles of *F. nucleatum*, *B. fragilis*, and *E. coli* in CRC susceptibility or progression are supported by preclinical studies and clinical sample-based studies. In view of the complex interactions between bacteria, these three samples are not the only microbes important for CRC, but they provide insight into targetable mechanisms of action in CRC. In the future, more research is needed to elucidate the association between gut microbiota and CRC.

## 3 Gut Microbiota Metabolites and Gastrointestinal Cancer

Despite various explorations into the interrelation between gut microbiota and cancer, the exact mechanisms of this interaction are still unclear. It has been reported that this interplay may be related to bacterial metabolites. Here, we focused on short-chain fatty acids (SCFAs), one of the most important gut microbiota metabolites, and discussed the current studies on the association between SCFAs and gastrointestinal cancer.

Moreover, gut microbiota can produce carbohydrate-active enzymes that ferment non-digestible carbohydrates, such as xylans, cellulose, and inulin, to generate SCFAs (95, 96). The great majority of SCFAs are the final products of bacterial fermentation, and the endogenous synthesis of the host is always trivial (97). SCFAs play multiple roles in human health and disease, such as inflammatory bowel disease, CRC, inflammatory bowel disease, diabetes, and atherosclerosis (98–100), while researchers have mainly focused on the effect of SCFAs on CRC (101–103). The gut microbiota has been revealed to produce approximately 50–100 mmol/L/day of SCFAs, mainly including acetate (C2), propionate (C3), and butyrate (C4) acid, and their ratio is 3:1:1 (104, 105). Acetate and propionate are formed by *Bacteroidetes*, whereas *Firmicutes* produce butyrate (106). Among these SCFAs, butyrate is deemed a crucial metabolite, which mediates the tumor-repressive effect of dietary fiber on CRC (105, 107, 108).

**Figure 3:**
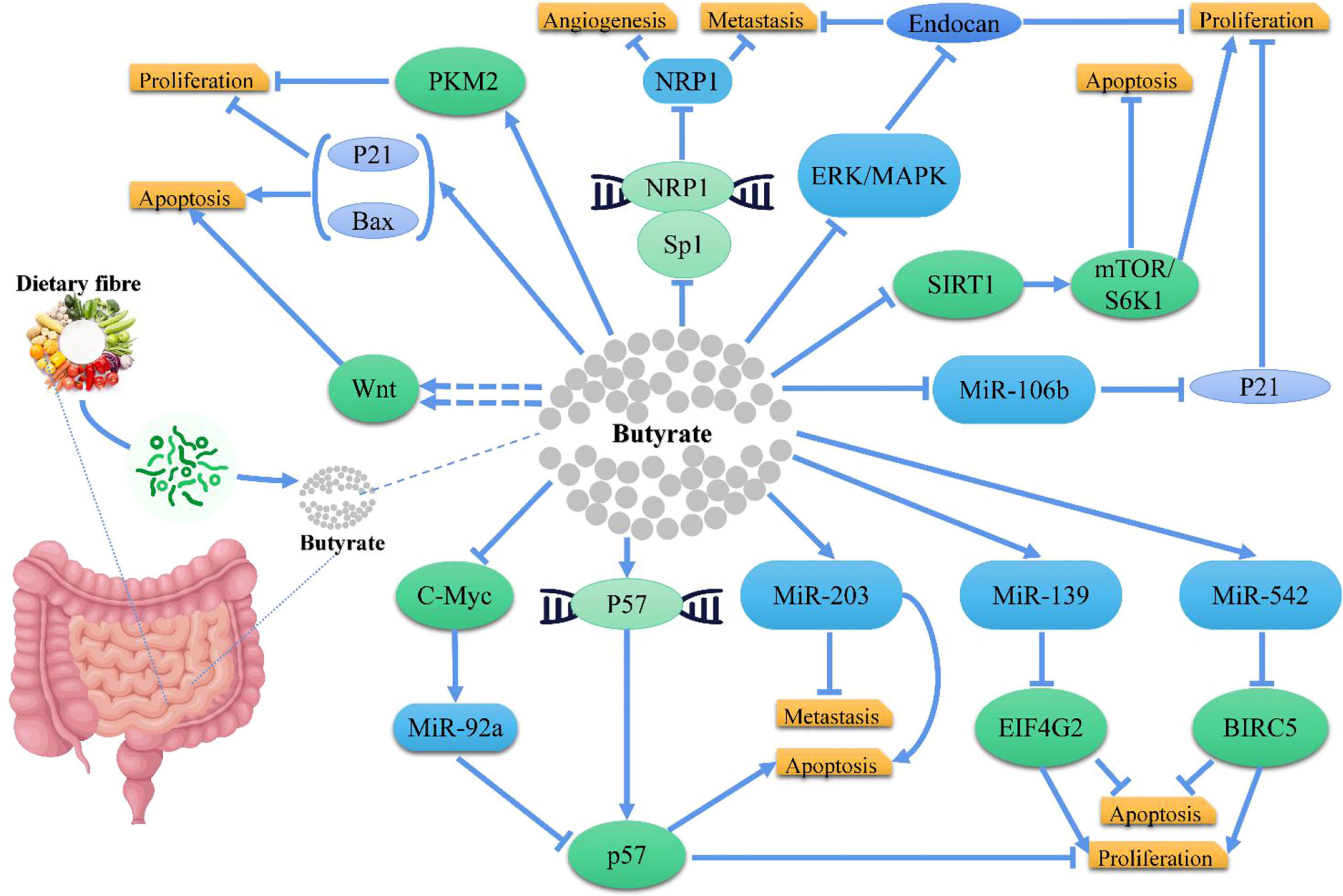
Butyrate involved in mechanisms and signaling pathway for development of CRC.

In CRC cells, butyrate inhibits histone deacetylases to increase the expression of genes that slow down the cell cycle and induce apoptosis (95, 96, 109). For example, Gamet et al. investigated the effects of SCFAs on the growth of the human adenocarcinoma cell line, HT29, and found that both butyrate and propionate inhibited the growth of HT29 cells, whereas acetate had no significant effect (110); Similarly, Hinnebusch et al. revealed that propionate, butyrate, and valerate suppressed the progression of colon carcinoma cells, while acetate and caproate had no effects (111); Zeng et al. indicated that butyrate had a greater inhibitory efficacy over propionate and acetate against CRC cell proliferation (112). Mechanistically, studies confirmed that butyrate inhibited CRC through different pathways (**Figure 3**), like Encarnação et al. suggested that butyrate inhibited the proliferation of CRC cells by regulating P21, and induced apoptosis by modulating BAX/BCL-2 ratio (113); Cao et al. demonstrated that butyrate treatment significantly inhibited proliferation and induced apoptosis in HCT116 cells with an increased BAX/BCL-2 ratio in CRC cells, and suggested that butyrate functioned *via* the deactivation of mTOR/S6K1 signaling mediated partly by SIRT1 downregulation (114); Yu et al. demonstrated that butyrate suppressed the expression of neuropilin I (NRP1) in colorectal cell lines through inhibition of Sp1 transactivation, and suppressed tumor cell migration and survival (115); Zuo et al. suggested that butyrate suppressed proliferation and migration in CRC cells through upregulating endocan expression *via* ERK2/MAPK signaling pathway (116); Chen et al. showed that *Clostridium butyricum* (one of the commonly used butyrate-producing bacteria in clinical settings) could inhibit intestinal tumor development by suppressed the Wnt/B-catenin signaling pathway, and activated G-protein coupled receptors (117); Bordonaro et al. evaluated that butyrate hyperactivated Wnt signaling, resulting in CRC cell apoptosis (118); Cucciolla et al. observed that butyrate upregulated p57 level by enhancing its transcription (119); In another research, Hu et al. identified a novel cellular mechanism that butyrate inhibited miR-92a transcription by reducing c-Myc, thus augmenting p57 level (120). Additionally, Li et al. demonstrated that butyrate suppressed the proliferation of CRC cells, through activating PKM2 *via* promoting its dephosphorylation and tetramerization, and thereby reprogramed the metabolism, inhibiting the Warburg effect while favoring energetic metabolism (121). Furthermore, Yoo et al. suggested that chronic exposure to butyrate induced butyrate resistance in CRC cells by triggering protective autophagy through the downregulation of AMPK/ACC and activation of Akt/mTOR signaling (122). Notably, miRNA expression is also intently related to the occurrence, development, and metastasis in CRC cells (123) and the expression of miRNA may be regulated by dietary factors, such as butyrate, like Han et al. showed that butyrate induced CRC cell apoptosis and inhibited the proliferation and invasion *via* upregulating miR-203 level (124); Ali et al. found that miR-139 and miR-542 acted cooperatively with butyrate to reduce CRC cell proliferation and induce apoptosis by regulating target genes, EIF4G2 and BIRC5 (125); Hu et al. concluded that butyrate regulated p21 expression *via* downregulating miR-106b level (126).

Although most of the available studies suggest that butyrate has an anti-tumor effect, it has also been observed to have a tumor-promoting effect in the development of CRC. This difference has been termed as the butyrate paradox and suggests that the effects of butyrate depend on its concentration, which is characterized as a high concentration of butyrate inhibiting tumorigenesis, while its low concentration promotes tumor progression (105). In more detail, on the one hand, Okumura et al. found that butyrate promoted tumorigenesis in CRC patients, and this colorectal tumorigenesis may be due to butyrate-induced senescence (127). Such effects were observed in many other studies (8, 105); however, the underlying mechanisms are still unclear. Thus, further studies are needed to understand the interplay between host genetics, microbial composition, and the presence of other gut metabolites, thereby clarifying this paradoxical result.

In conclusion, while many studies on SCFA and their association with cancer have been conducted, CRC has received the most attention. Further research is needed to explore the role of SCFA in other cancers, such as GC, pancreatic cancer, and hepatic carcinoma, to determine the exact role of SCFA in cancer development and treatment. In addition, in-depth studies are needed to investigate the effect of SCFAs on the efficacy and safety of chemotherapy or immunotherapy and their correlation with cancer prognosis.

## 4 Effects of Gut Microbiota on Gastrointestinal Cancer Treatment

With the increasing knowledge of gut microbiota, researchers have gained a deeper understanding of the impact of abnormal bacterial metabolism on the host. Numerous studies have shown that microbiota can metabolize, activate, and inactivate many common drugs, while their impact on cancer treatment has only received insufficient attention until recently (128, 129). Here, we have focused on the relevance of microbiota to chemotherapy, immunotherapy, and microbiota modulation.

### 4.1 Microbiota and Chemotherapy

The gut microbiota is metabolically active, while many cancer chemotherapeutic drugs act in an anti-metabolism manner. Thus, there is a potential interaction between gut microbiota and chemotherapy. Growing evidence suggests that gut microbiota can influence the efficacy of cancer treatment. For example, certain gammaproteobacteria can metabolize gemcitabine into an inactive form, thus inducing gemcitabine resistance (130); gemcitabine resistance was abrogated by the co-treatment with ciprofloxacin in a CRC mouse model (130); and Yuan et al. also suggested that antibiotics disrupt the gut microbiota in mice and reduce 5-fluorouracil efficacy (131). Similarly, Iida et al. demonstrated that oxaliplatin and cisplatin treatment exhibited reduced antitumor efficacy and survival in various tumor-bearing mice in the presence of antibiotics (132). Additionally, Yu et al. demonstrated that *Fusobacterium* plays a critical role in mediating CRC chemoresistance by activating autophagy (133). Furthermore, the treatment of CRC xenograft-bearing mice with the antibiotic metronidazole reduced *Fusobacterium* load, cancer cell proliferation, and overall tumor growth (134). Although most of the current research is derive from preclinical experiments, we strongly believe that the gut microbiota is closely related to chemotherapy.

### 4.2 Microbiota and Immunotherapy

Increasing evidence has highlighted that gut microbiota is involved in the clinical response to cancer immunotherapy (135–137). These immunotherapies mainly target programmed cell death protein-1 (PD-1) and cytotoxic T lymphocyte-associated antigen-4 (CTLA-4) blockades (138). A study in 2015 confirmed that gut microbiota could modulate the effect of anti-PD-1/PD-L1 monoclonal antibodies in mice (139), and commensal *Bifidobacterium* enhanced the immunotherapeutic effects by activating dendritic cells for CD8+ T-cell priming and infiltration in the tumor microenvironment (139). In addition, Rizvi et al. showed that *Bifidobacterium* enhanced the efficacy of anti-PD-1 monoclonal antibodies in mice with melanoma by secreting the metabolite hippurate and inhibiting PD-1 expression (140); Sun et al. found that *Bifidobacterium* altered the composition of the gut microbiota in a manner dependent on regulatory T cells; this altered commensal community enhanced both the mitochondrial fitness and the IL-10-mediated suppressive functions of intestinal Tregs, contributing to the reduction of colitis during immune checkpoint blockade (141); and Wang et al. suggested that *Bifidobacterium* could mitigate intestinal immunopathology induced by CTLA-4 blockade (142). However, it remains unknown whether *Bifidobacteria* can reduce virulence while enhancing efficacy. Other gut bacteria besides *Bifidobacterium* may also modulate immunotherapy. Like Si et al. demonstrated, oral administration of live *Lactobacillus rhamnosus* GG augmented the antitumor activity of anti-PD-1 by increasing tumor-infiltrating dendritic and T cells (143). Mechanistically, treatment with live *Lactobacillus rhamnosus* GG triggered type I interferon production in dendritic cells, enhancing the cross-priming of antitumor CD8+ T cells (143); Vétizou et al. found that the antitumor effects of CTLA-4 blockade depended on distinct bacteroide species (144). Numerous studies have determined various microbial species related to the response of immune checkpoint inhibitors (135, 145–148). However, it remains unknown whether their interactions with one another and which microbes are more important in immune checkpoint inhibitors (149).

### 4.3 Microbiota Modulation

The microbiota modulation strategy is largely based on the regulatory role of specific gut microbiota in anti-tumor immunity. The principle of this strategy is to facilitate the effect of immune-enhancing gut microbiota while reducing the effect of immunosuppressive gut microbiota.

#### 4.3.1 Prebiotics and Probiotics

Many clinical trials have been conducted to investigate the effect of prebiotics or probiotics on tumor therapy, some of which reported improved clinical outcomes in patients receiving probiotics, while others were not significantly different (150–153). Future studies may need to increase the number of samples and tumor types and standardize research methods to further clarify the impact of prebiotics and probiotics in tumor treatment. Notably, the limited evidence indicates that probiotic strains would not be the most suitable strains to treat tumors.The concept of prebiotics was first proposed in 1995 and redefined in December 2016 (154, 155). Prebiotics are defined by the International Scientific Association for Probiotics and Prebiotics as “substrates that are selectively utilized by host microorganisms to confer health benefits to the host” (155). The two important groups of prebiotics are fructo-oligosaccharides (FOS) and galacto-oligosaccharides (GOS) (156). FOS is naturally present in asparagus, bananas, chicory root, garlic, and onion, as well as synthesized commercially (155, 157); GOS is produced commercially from lactose by B-galactosidase (156). The effect of FOS and GOS on gut microbiota modulation has been shown previously (157). In addition, the effects of prebiotics have been shown to engage in tumor therapy, such as: Taper et al. suggested that treatment with inulin or oligofructose potentiated the effects of cancer therapy (158, 159); Dewulf et al. confirmed that treatment with inulin or oligofructose led to subtle changes in gut microbiota (160); *Escherichia coli* Nissle 1917 (EcN), a genetically tractable probiotic with a well-established human safety record, is emerging as a preferred chassis (161). In theory, prebiotics can selectively enrich beneficial probiotics and increase SCFA production. However, how prebiotics increase the effects of chemotherapy and immunotherapy requires further research (139, 162, 163).

Probiotics are living microorganisms, mainly including *Lactobacillus* and *Bifidobacterium*, can confer health benefits on the host at a certain concentration (164). Botta et al. suggested that *Lactiplantibacillus* plantarum inhibited colon cancer cell proliferation by butyrogenic capability (165). Do Carmo et al. confirmed that *Propionibacterium freudenreichii* alleviated mucositis induced by 5-fluorouracil chemotherapy (166); Sivan et al. suggested that *Bifidobacterium* promoted antitumor immunotherapy effects of anti-PD-L1 (139); Si et al. found that *Lactobacillus rhamnosus* GG improved response to immune checkpoint blockade (143). However, a recent study indicated an impaired-treatment response to anti-PD-1 therapy in mice receiving a low-fiber diet or probiotics, and that in the tumor microenvironment there is a lower frequency of interferon-γ-positive cytotoxic T cells (167). Compared to previous preclinical studies, this mechanistic study clarified the opposite results. Most of the current clinical studies have only shown the effect of probiotics on gut microbiota, but not clarified their impact on immunotherapy (146, 148, 168).

#### 4.3.2 Fecal Microbiota Transplantation (FMT)

FMT is another clinical strategy for manipulating gut microbiota and has been approved by the FDA for treating *Clostridium difficile* infection (149). FMT can transfer entire fecal microbial community, namely, bacteria, fungi, viruses, and their metabolites, from a healthy donor to a recipient (149, 169). Wang et al. first reported a successful case series of immune checkpoint inhibitors-associated colitis treated with FMT (170), in which two patients reconstituted the gut microbiota, and a substantial reduction in CD8+ T-cell density with a concomitant increase in CD4+ FoxP3+ was observed within the colonic mucosa, offering a potential mechanism through which FMT could abrogate ICI-associated toxicity. However, certain limitations existed in this case, and more clinical trials are needed to evaluate the effect of this approach and further elucidate the underlying mechanisms. Recently, FMT has begun to be inspected in combination with checkpoint blockade therapy, as Routy et al. found that the clinical benefit of immune checkpoint inhibitors (ICIs) was attenuated in advanced cancer patients treated with antibiotics (135). Furthermore, FMT from cancer patients who responded to ICIs into antibiotic-treated or germ-free mice improved the effects of PD-1, whereas FMT from nonresponding patients failed (135). More recently, clinical trials of FMT in combination with checkpoint blockade therapy are ongoing or completed, like Matson et al. reviewed several clinical trials evaluating the potential of FMT to enhance immune checkpoint blockade therapy, primarily in patients with metastatic melanoma (171), implicating the feasibility and safety of FMT in cancer treatment. Notably, a clinical trial of FMT capsule for improving the efficacy of anti-PD-1 among patients with gastrointestinal cancer is recruiting (NCT04130763, https://clinicaltrials.gov). Additionally, Kassan et al. evaluated the suitability of stool from candidate FMT donors for clinical use and suggested that only 3% of donors would pass such quality control assessments (172). For solving such issue, more studies have been conducted and shown that a good given FMT donor should have the necessary gut microbiota composition to correct the microbiota deficiency in one patient but not another (173–178). Furthermore, unlike other microbiota modulation methods, the effects of FMT could last for more than 24 weeks, thus frequent interventions are not required (149, 169). With further studies on the mechanisms by which the gut microbiota modulates host antitumor immunity, and further confirmation of the functions of related bacteria, we believe that the selection of FMT donors for specific patients will be more convenient and faster in the future.

#### 4.3.3 Antibiotics

Bacterial depletion by antibiotics is another strategy for manipulating the gut microbiota. Prophylactic antibiotics are always given with chemotherapy or immunotherapy to prevent potentially life-threatening infections from immunosuppression caused by chemotherapy and immunotherapy.

As previously mentioned, certain gammaproteobacteria could induce gemcitabine resistance, and antibiotics targeting gammaproteobacteria-improved gemcitabine response in patients with pancreatic ductal adenocarcinoma (130); however, Wu et al. found that antibiotic administration reduced chemotherapy efficacy and was associated with poor prognosis in patients with esophageal cancer (179); And Nenclares et al. suggested that antibiotic therapy was associated with a negative outcome in locally advanced head and neck cancer (180). Notably, Zheng et al. showed that oral or intravenous administration of irinotecan-loaded dextran nanoparticles covalently linked to azide-modified phages that inhibit the growth of *F. nucleatum* significantly improves the efficiency of chemotherapy in CRC mice (181). These results suggest that using antibiotics with chemotherapy is a double-edged sword. Therefore, more differentiated strategies can be applied, such as selective use of antibiotics or targeted delivery of antibiotics. With the advances in nanotechnology, targeted-delivery of antibiotics may be possible to balance the risks and benefits of prophylactic antibiotic use in cancer chemotherapeutic patients.

Researchers have observed reduced response to ICIs in patients treated with antibiotics in preclinical and clinical trials. For example, Vétizou et al. found that tumors did not respond to CTLA blockade in antibiotic-treated or germ-free mice (144); Pinato et al. suggested that gut dysbiosis caused by broad-spectrum antibiotic therapy-impaired ICIs response (182); Tinsley et al. highlighted that reduced clinical benefit from ICIs was associated with antibiotic use in advanced cancer (183). Similarly, Derosa et al. found that antibiotics were associated with worse treatment outcomes of ICIs in non-small cell lung cancer and renal cell carcinoma (184). Thus, modulation of ATB-related dysbiosis and gut microbiota composition may be a strategy to improve clinical outcomes with ICIs, as Wilson et al. confirmed that overall survival and progression-free survival are longer in patients who have not received antibiotics compared to patients with antibiotic use (185). Furthermore, antibiotic use 42 days before starting ICIs appears most harmful to the outcome, while antibiotic use in the 60 days before starting ICIs appears to have no significant difference in outcome (185). Palleja et al. found that it takes time for gut microbiota to recover in healthy adults after antibiotic exposure (186). We speculate that it takes more time for gut microbiota to recover in tumor patients after antibiotic exposure. Thus, antibiotics treatment should be avoided before ICIs (149, 187, 188). Perhaps, it is a better strategy using probiotics or FMT to modulate gut microbiota before ICIs other than using antibiotics.

## 5 Perspectives and Future Directions

Humans harbor trillions of resident microorganisms, which make up the microbiota. Microbiota play a vital role in various aspects of human health and disease. In recent years, an increasing number of studies have focused on the impact of gut microbiota on host metabolism and disease. Here, we reviewed the potential roles of gut microbiota and gut microbial metabolites in gastrointestinal cancer and explored the therapeutic potential of gut microbiota in gastrointestinal cancer. However, in terms of the impact of gut microbiota on tumorigenesis, development, and treatment, our knowledge may only be the tip of the iceberg, and there are still many problems to be solved.

Firstly, can cancer clusters be explained by microbiota? Cancer cluster is defined as the number of cancer cases occurring in a group of people over a specific period time in a specific geographic area that is higher than expected (189), for example, East Asia is a hotspot for gastric cancer incidence and mortality in this world; Linqu, Shandong Province, is a region with the highest incidence and mortality rate of GC in China (28, 190); and in sub-Saharan Africa, there is a substantial, early-onset CRC has increased significantly (191, 192). However, the specific factors contributing to the occurrence of these cancers are unclear, and further research is needed to determine whether gut microbiota is associated with increased cancer incidence in these populations. Second, the composition of gut microbiota is affected by age, diet, genetics, lifestyle, and medical conditions, are these factors also needed to be monitored? These factors are closely related to geographical location, so is it more meaningful to incorporate geographic information into microbiota studies and cancer detection? Third, what studies do researchers need to explore the relationship between gut microbiota and pathogenesis as well as treatment of gastrointestinal cancer? What is the ideal way to use gut microbes for gastrointestinal cancer treatment? Finally, in order to identify a reliable gut microbiota for predictable gastrointestinal cancer risk and patient prognosis, or for gut microbiota-based treatment strategies, we need standardized approaches and uniform designs for gut microbiota sampling in large and diverse patient populations, which requires our joint efforts.

## Author Contributions

QG, HQ, XL, XZ, TQ, and LC reviewed the literature and drafted the article. ZC organized figures and tables. QG, HQ, and WZ finalized the paper and provided suggestions to improve it. All authors participated in designing the concept of this manuscript. All authors listed have made a substantial, direct, and intellectual contribution to the work and approved it for publication.

## Funding

The study was supported by the Medical Science and Technology Research Project of Henan Province (no. SBGJ202003010), the Medical Science and Technology Research Project of Henan Province (no. LHGJ20190675), and the Doctoral Research Start-up Foundation of Henan Cancer Hospital.

## Conflict of Interest

The authors declare that the research was conducted in the absence of any commercial or financial relationships that could be construed as a potential conflict of interest.

## Publisher’s Note

All claims expressed in this article are solely those of the authors and do not necessarily represent those of their affiliated organizations, or those of the publisher, the editors and the reviewers. Any product that may be evaluated in this article, or claim that may be made by its manufacturer, is not guaranteed or endorsed by the publisher.

## Abbreviation

GC, gastric cancer; CRC, colorectal cancer; H. pylori, Helicobacter pylori; VacA, vacuolating cytotoxin A; CagA, cytotoxin-associated gene A; GAD, gastric adenoma; EGC, Early gastric cancer; AGC, Advanced gastric cancer; IM, gastritis without intestinal metaplasia; HC, healthy controls; SG, superficial gastritis; SRCC, signet-ring cell carcinoma; CG, non-atrophic chronic gastritis IN intraepithelial neoplasia; AG, atrophic gastritis; LAB, lactic acid bacteria; F. nucleatum, Fusobacterium nucleatum; B. fragilis, Bacteroides fragilis; E. coli, Escherichia coli; ETBF, Enterotoxigenic B. fragilis; SCFAs, short-chain fatty acids; PD-1, programmed cell death protein-1; CTLA-4, cytotoxic T lymphocyte-associated antigen-4; FMT, Fecal Microbiota Transplantation; ICIs, immune checkpoint inhibitors.
